# Seroprevalence of spotted fever rickettsiosis and ehrlichiosis among food processing workers and their families in Latino communities in North Carolina

**DOI:** 10.1371/journal.pgph.0005116

**Published:** 2025-09-02

**Authors:** Melissa K. Cutshaw, Michael Sciaudone, Brenda Vasquez-Martinez, Colleen M. McClean, Oksana Kharabora, Katherine Murray, Stephen Strohminger, Miriana Moreno Zivanovich, Rachel Gurnett, Emperatriz Morales Salgado, Ross M. Boyce, Natalie M. Bowman

**Affiliations:** 1 Department of Medicine, Duke University, Durham, North Carolina, United States of America; 2 Section of Infectious Diseases, Department of Medicine, Tulane University, New Orleans, Louisiana, United States of America; 3 School of Medicine, University of North Carolina at Chapel Hill, Chapel Hill, North Carolina, United States of America; 4 Facultad de Salud Publica y Administracion, Universidad Peruana Cayetano Heredia, Lima, Peru; 5 Department of Internal Medicine—Pediatrics, Baylor College of Medicine and Texas Children’s Hospital, Houston, Texas, United States of America; PLOS: Public Library of Science, UNITED STATES OF AMERICA

## Abstract

Workers in food processing industries are subject to many occupational health risks and disparities, but little is known about their risk of tickborne diseases. We examined a cohort of Latino individuals working in the meat packing, produce processing, and farming industries and their family members in central North Carolina, where incidence of tickborne infections is high. Blood samples were tested for IgG antibodies against Spotted Fever Group Rickettsiosis (SFGR) and *Ehrlichia chaffeensis*. Covariates of interest included age, sex, primary language, work industry, indoor vs. outdoor work, home characteristics, medical comorbidities, and travel history. Among 201 Latino food processing workers and their family members, the seroprevalence of SFGR and *Ehrlichia* was 14.9% and 19.9%, respectively. Almost a third of participants were seropositive for at least one infection. SFGR seropositive individuals were significantly older than seronegative individuals (median 45 [interquartile range 35–55] vs. 33 [14–45] years, p < 0.001), while *Ehrlichia* seropositivity appeared to have a bimodal distribution by age, with peaks in children under age 10 and adults in their forties and fifties. Farm workers had higher seroprevalence of SFGR (25.0%) than other workers (13.5%), although this did not reach statistical significance (p = 0.13). Having a seropositive household member for either infection was a risk factor for seropositivity for the same infection, adjusted for age and household clustering (adjusted OR [aOR] 8.26, 95% CI [confidence interval] 3.27-20.90 for SFGR; aOR 11.24, 95% CI 4.24-29.80 for *Ehrlichia*). Seroprevalence for SFGR and *Ehrlichia* was similar between index workers and household members when adjusted for age. Our findings indicate that Latino food processing communities in North Carolina have high exposure to tickborne disease, and older age and having seropositive household members are key risk factors.

## Introduction

Individuals in the meat packing, produce processing, and farm working industries, referred to collectively as food processing industries are exposed to some of the greatest occupational hazards in the United States [1–4]. These risks are related to physically strenuous work, long hours, dangerous machinery, chemical exposures, and poor work protections [2,3]. Furthermore, workers – many of whom are migrants - often have limited access to health care resources and little autonomy to advocate for improved working conditions. Health disparities among food processing workers were recently highlighted by the disproportionate burden in this population during the COVID-19 pandemic, particularly among racial and ethnic minorities [5]. Yet, little is known about the potential occupational risk of tickborne diseases in food processing workers.

Over the last twenty years, the annual incidence of tickborne diseases has more than doubled in the U.S., and workers in certain industries appear to be more susceptible, particularly those requiring work outdoors [6–8]. In North Carolina, two of the most common tickborne diseases are Spotted Fever rickettsiosis (SFR), a category which includes Rocky Mountain spotted fever (RMSF, caused by *Rickettsia rickettsii*), and ehrlichiosis. Transmitted by the American dog tick (*Dermacentor variabilis)* and lone star tick (*Amblyomma americanum)*, respectively, both infections can be fatal if not promptly identified and treated [9]. According to the North Carolina Department of Health and Human Services, the average incidence rate in North Carolina from 2015-2019 was 0.65 cases per 100,000 residents for ehrlichiosis, and 5.15 cases per 100,000 residents for SFR, over twice the national average [10].

Our study group previously conducted an observational cohort study among Latino workers employed in the food processing industry and their family members in central North Carolina, which has one of the largest food processing industries in the U.S [11]. Although the primary aim was to investigate the epidemiology of COVID-19, we also examined associations between other infections, including tickborne diseases, with potential occupational risk factors. By leveraging the existing database of food processing workers and their family members, we investigated sociodemographic and occupational risk factors for *Ehrlichia* and Spotted Fever Group Rickettsiosis (SFGR) infection among Latino food processing communities in North Carolina.

## Methods

### Study population

We conducted a secondary analysis of the COFF-NC (COVID-19 in Farming and Food processing industries in North Carolina) study. This cohort of 224 participants aimed to study the epidemiology of COVID-19 and other infections related to occupational exposures for food processing workers in North Carolina. The study was conducted in central North Carolina in an area with a large number of workers employed in food processing. Recruitment of study participants occurred between September 1 and December 31, 2020. Adults aged ≥18 years who had worked for at least 2 weeks in a meat packing plant, food processing facility or commercial farm from 1 February 2020 and resided in North Carolina were recruited and classified as index workers. Household members of index workers aged ≥12 months were invited to participate. Latino ethnicity and industry were identified by self-reported data. North Carolina has a large population of migrant farm workers, primarily through the H-2A Agricultural Visa Program; these individuals were identified by self-reported data [12]. Data and specimen collection techniques have been previously reported [13,14].

### Serology

Plasma samples were tested by a commercial indirect fluorescence immunoassay (IFA) for detection of IgG specific for *Ehrlichia chaffeensis* (Fuller Laboratories, CA, USA) [15]. The manufacturer’s protocol was followed. Briefly, each sample was diluted at 1:64 in Phosphate-Buffered saline (PBS) at pH 7.2. Negative control was used with no dilution and a dilution gradient of the positive control diluted in PBS until 1:1024 dilution was used with each batch of samples tested. Samples at 1:64 dilution with the presence of fluorescence were retested to confirm positivity. Samples without the presence of fluorescence at 1:64 dilution were reported as negative.

A commercial SFGR IgG indirect enzyme immunoassay (ELISA) antibody kit was used to test all plasma samples (Fuller Laboratories, CA, USA) [15]. As stated in the manufacturer protocol, samples were diluted 1:100 in sample diluent. Positive control, negative control, and cutoff calibrator were diluted 1:10 in sample diluent. The index value was obtained by dividing the absorbance of the samples by the mean absorbance value of the cutoff calibrator. Samples with index values from 0.9 to 1.1 were reported as equivocal, samples with index value >1.1 were reported as positive, and samples with index value <0.9 were reported as negative.

### Statistical analysis

Two-sample t-tests, chi-square tests, Fisher’s exact test, and the Wilcoxon rank-sum test were used to compare participant and household characteristics between groups. Equivocal results for SFGR seroprevalence were considered negative results; sensitivity analyses were performed in which these results were considered positive or excluded (Supplement). We employed binary logistic regression models to calculate odds ratios for a primary outcome of SFGR or *Ehrlichia* seropositivity by covariates of interest, including age, sex, primary language, industry, employment type (full-time, part-time, unemployed, or other), medical comorbidities (diabetes or hypertension, history of smoking tobacco products, or regular alcohol use defined as ≥7 drinks per week), school attendance, and recent travel (defined as travel within the last three weeks in which at least one night was spent away from home). Among workers, covariates also included indoor vs. outdoor work, average hours worked per week, being a migrant farm worker, home type, and household size. Given the marked association between age and seropositivity, a post-hoc analysis was performed in which covariates significantly associated with seropositivity were further assessed by logistic regression adjusted for age. We used generalized estimating equations to account for potential clustering of cases within households. Statistical analyses were performed in Stata 18.0 (StataCorp, College Park, MD).

### Ethics

This study was approved by the Institutional Review Board at the University of North Carolina at Chapel Hill (IRB 20-2032). Written informed consent was obtained from participants in English or Spanish by bilingual study personnel. Participant confidentiality was maintained by removing personal identifiers from datasets and biospecimens. Participant data with personal identifiers were stored in a locked cabinet only accessible to authorized study personnel. Electronic participant data did not include identifiers and were only accessible through a password-protected REDCap database to authorized study personnel.

### Inclusivity in global research

Additional information regarding the ethical, cultural, and scientific considerations specific to inclusivity in global research is included in the Supporting Information (S1 Checklist).

## Results

### Baseline characteristics

Sufficient plasma were available for testing for 201 of 224 (89.7%) individuals enrolled in COFF-NC, including 112 index workers and 89 family members. Participants excluded due to inadequate sample volume did not significantly differ from the remaining participants by age or sex. Participants had a median age of 34 years (interquartile range [IQR] 15–47), 43.8% were male, and 80.6% spoke Spanish as their primary language. Slightly less than half of participants were employed full-time, and about a quarter attended school full-time (Table 1). Among index workers, 42 worked in meat packing, 10 worked in produce processing, and 60 worked in farming. Of the farm workers, 9 were self-reported as migrant farm workers.

**Table 1:** Baseline characteristics.

Variable	Index workers	Family members	Overall
N	112	89	201
Sociodemographic characteristics			
Age (years), median (interquartile range)	43 (33-49.5)	14 (10-33)	34 (15-47)
Sex			
Female	71 (63.4%)	42 (47.2%)	113 (56.2%)
Male	41 (36.6%)	47 (52.8%)	88 (43.8%)
Primary language			
English	9 (8.0%)	30 (33.7%)	39 (19.4%)
Spanish	103 (92.0%)	59 (66.3%)	162 (80.6%)
Employment			
Industry			
Meat packing	42 (37.5%)	–	42 (20.9%)
Produce processing	10 (8.9%)	–	10 (5.0%)
Farming	60 (53.6%)	–	60 (29.9%)
Employment type			
Currently employed full-time	81 (72.3%)	15 (16.9%)	96 (47.8%)
Currently employed part-time	12 (10.7%)	3 (3.4%)	15 (7.5%)
Unemployed	18 (16.1%)	17 (19.1%)	35 (17.4%)
Other	1 (0.9%)	0 (0.0%)	1 (0.5%)
Medical comorbidities			
Diabetes mellitus or hypertension	34 (30.3%)	7 (7.9%)	41 (20.4%)
Ever smoker	13 (11.6%)	5 (5.6%)	18 (9.0%)
Regular alcohol use^a^	5 (4.5%)	2 (2.2%)	7 (3.5%)
Social characteristics			
School attendance	5 (4.5%)	53 (60.0%)	58 (28.9%)
Recent travel^b^	3 (2.7%)	11 (12.4%)	14 (7.0%)
Tickborne disease			
SFGR seropositive	22 (19.6%)	8 (9.0%)	30 (14.9%)
*Ehrlichia* seropositive	25 (22.3%)	15 (16.9%)	40 (19.9%)
Either SFGR or *Ehrlichia* seropositive	42 (37.5%)	21 (23.6%)	63 (31.3%)
Both SFGR and *Ehrlichia* seropositive	5 (4.5%)	2 (2.2%)	7 (3.5%)

^a^Defined as routinely drinking 7 or more drinks per week.

^b^Defined as travel within the last 3 weeks in which at least one night was spent away from home.

SFGR: Spotted Fever Group Rickettsiosis.

### SFGR seroprevalence

**Fig 1:**
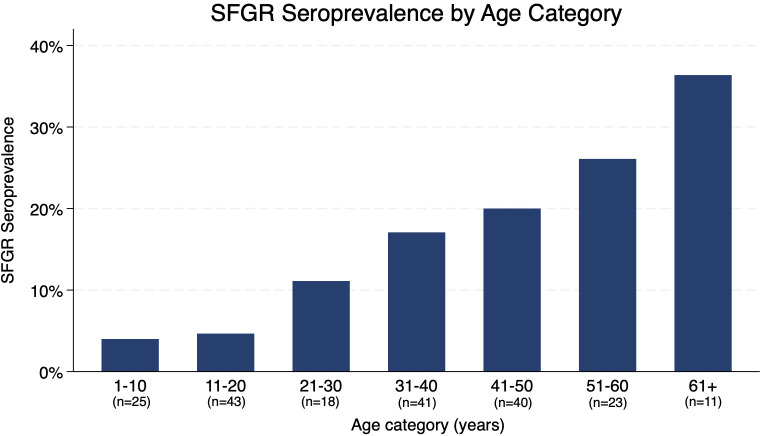
SFGR seroprevalence by age category.

Among 201 total participants, 30 (14.9%) were seropositive for SFGR (Table 2). SFGR seropositive participants were significantly older than seronegative participants (median 45 [IQR 35–55] vs. 33 [14–45] years, p < 0.001) (Fig 1). Older participants had 1.05 times the odds of SFGR positivity (95% confidence interval [CI] 1.02-1.07). Participants with a smoking history (odds ratio [OR] 3.19, 95% confidence interval [CI]: 1.09-9.30) or with current school attendance (OR 0.15, 95% CI: 0.03-0.66) had higher and lower odds of SFGR seropositivity, respectively; these associations lost statistical significance when controlled for age. No other participant factors were significantly associated with SFGR seropositivity, including sex, primary language, employment status, medical comorbidities, or recent travel. Among adults aged 18 years and older, SFGR seropositivity rates did not significantly differ between index workers and household members.

**Table 2:** Participant characteristics associated with SFGR seropositivity among index workers and household members.

Variable	Seropositive	Seronegative	P-value	OR	aOR
N	30	171			
Sociodemographic characteristics					
Age (years), median (interquartile range)	45 (35-55)	33 (14-45)	<0.001	**1.05 (1.02-1.07)**	
Sex			0.39	0.71 (0.32-1.58)	
Female	19 (63.3%)	94 (55.0%)			
Male	11 (36.7%)	77 (45.0%)			
Primary language			0.06	3.87 (0.88-17.0)	
English	2 (6.7%)	37 (21.6%)			
Spanish	28 (93.3%)	134 (78.4%)			
Employment status			0.55	1.01 (0.63-1.62)	
Currently employed full-time	19 (63.3%)	77 (45.0%)			
Currently employed part-time	1 (3.3%)	14 (8.2%)			
Unemployed	8 (26.7%)	27 (15.8%)			
Other	0 (0.0%)	1 (0.6%)			
Medical comorbidities					
Diabetes mellitus or hypertension	8 (26.7%)	33 (19.3%)	0.36	1.52 (0.62-3.72)	
Ever smoker	6 (20.0%)	12 (7.0%)	0.03	**3.19 (1.09-9.30)**	2.09 (0.68-6.48)^a^
Regular alcohol use^c^	3 (10.0%)	4 (2.3%)	0.04	4.64 (0.98-21.9)	
Social characteristics					
School attendance	2 (6.7%)	56 (32.7%)	0.005	**0.15 (0.03-0.66)**	0.43 (0.07-2.54)^a^
Recent travel^d^	0 (0.0%)	14 (8.2%)	0.11	–	
Household analysis					
Household member seropositive for SFGR	22 (73.3%)	47 (27.5%)	<0.001	7.26 (3.02-17.42)	8.26 (3.27-20.90)^b^

^a^Adjusted for age.

^b^Adjusted for age and household clustering.

^c^Defined as routinely drinking 7 or more drinks per week.

^d^Defined as travel within the last 3 weeks in which at least one night was spent away from home.

SFGR: Spotted Fever Group Rickettsiosis.

Among 112 index workers, 22 participants (19.6%) were seropositive for SFGR (Table 3). SFGR seropositive participants were significantly older than seronegative participants (median 45.5 [IQR 37–52] vs. 41 [31–49] years, p = 0.05). SFGR seropositivity rates were highest among farm workers (15, 25.0%), followed by meat packing workers (7, 16.7%) and produce processing workers (0, 0.0%), although no statistically significantly differences were seen between groups. There was no significant association with indoor vs. outdoor work, hours worked per week, home type, or household size. In a sensitivity analysis in which equivocal SFGR results (n = 6) were considered either positive or excluded, no significant change in results was found (S1 and S2 Tables).

**Table 3:** Participant characteristics associated with SFGR seropositivity among index workers.

Variable	Seropositive	Seronegative	P-value
N	22	90	
Sociodemographic characteristics			
Age (years), median (interquartile range)	45.5 (37-52)	41 (31-49)	0.05
Sex			0.98
Female	14 (63.6%)	57 (63.3%)	
Male	8 (36.4%)	33 (36.7%)	
Primary language			0.50
English	1 (4.5%)	8 (8.9%)	
Spanish	21 (95.5%)	82 (91.1%)	
Employment Industry			
Meat packing	7 (31.8%)	35 (38.9%)	0.54
Produce processing	0 (0.0%)	10 (11.1%)	0.10
Farming	15 (68.2%)	45 (50.0%)	0.13
Primarily indoor work	15 (68.2%)	55 (61.1%)	0.54
Average hours worked per week	40.9 ± 9.1	40.9 ± 10.5	1.00
Migrant farm worker	2 (9.1%)	7 (7.8%)	0.77
Home characteristics			
Home type			0.86
Single family	6 (27.3%)	25 (27.8%)	
Apartment	0 (0.0%)	1 (1.1%)	
Duplex/townhouse	0 (0.0%)	1 (1.1%)	
Trailer	16 (72.7%)	60 (66.7%)	
Dorm	0 (0.0%)	3 (3.3%)	
Household size	3.64 ± 1.7	4.42 ± 4.1	0.06

SFGR: Spotted Fever Group Rickettsiosis.

### *Ehrlichia* seroprevalence

**Fig 2:**
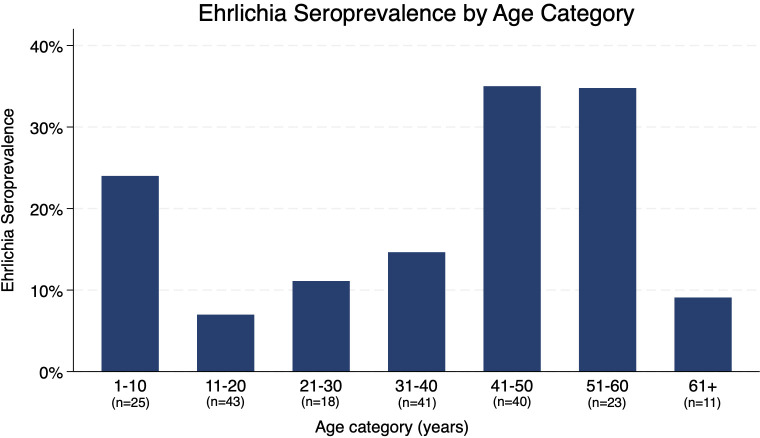
*Ehrlichia* seroprevalence by age category.

Among 201 total participants, 40 (19.9%) were seropositive for *Ehrlichia* (Table 4). *Ehrlichia* seropositivity appeared to have a bimodal association with age, with peaks in children under age 10 and adults in their forties and fifties (Fig 2). Participants with a history of diabetes mellitus or hypertension had higher odds of *Ehrlichia* seropositivity (OR 2.67, 95% CI: 1.24-5.77), and those who currently attended school had lower odds of *Ehrlichia* seropositivity (OR 0.37, 95% CI: 0.14-0.93); these associations lost statistical significance when controlled for age. No other participant factors were significantly associated with *Ehrlichia* seropositivity, including sex, primary language, employment status, or recent travel.

**Table 4:** Participant characteristics associated with *Ehrlichia* seropositivity among index workers and household members.

Variable	Seropositive	Seronegative	P-value	OR	aOR
N	40	161			
Sociodemographic characteristics					
Age (years), median (interquartile range)	43.5 (28-50)	33 (14-44)	0.07	1.02 (1.00-1.04)	
Sex			0.86	0.94 (0.47-1.89)	
Female	23 (57.5%)	90 (55.9%)			
Male	17 (42.5%)	71 (44.1%)			
Primary language			0.09	2.50 (0.83-7.50)	
English	4 (10.0%)	35 (21.7%)			
Spanish	36 (90.0%)	126 (78.3%)			
Employment status			0.58	1.28 (0.83-1.97)	
Currently employed full-time	18 (45.0%)	78 (48.4%)			
Currently employed part-time	4 (10.0%)	11 (6.8%)			
Unemployed	10 (25.0%)	25 (15.5%)			
Other	0 (0.0%)	1 (0.1%)			
Medical comorbidities					
Diabetes mellitus or hypertension	14 (35.0%)	27 (16.8%)	0.01	**2.67 (1.24-5.77)**	2.34 (0.86-6.35)^a^
Ever smoker	3 (7.5%)	15 (9.3%)	0.67	0.76 (0.21-2.75)	
Regular alcohol use^d^	2 (2.5%)	5 (3.1%)	0.56	1.64 (0.31-8.79)	
Social characteristics					
School attendance	6 (15.0%)	52 (32.3%)	0.03	**0.37 (0.14-0.93)**	0.44 (0.13-1.46)^a^
Recent travel^d^	0 (0.0%)	14 (8.7%)	0.06	–	–
Household analysis					
Household member seropositive for *Ehrlichia*	34 (85.0%)	65 (40.4%)	<0.001	8.37 (3.32-21.07)	11.24 (4.24-29.80)^b^

^a^Adjusted for age.

^b^Adjusted for age and household clustering.

^c^Defined as routinely drinking 7 or more drinks per week.

^d^Defined as travel within the last 3 weeks in which at least one night was spent away from home.

Among 112 index workers, 25 (22.3%) were seropositive for *Ehrlichia* (Table 5). *Ehrlichia* seroprevalence did not significantly differ by industry. *Ehrlichia* seroprevalence was similar between industries (23.8% among meat packing workers, 20.0% among produce processing workers, and 21.7% among farm workers). There was no significant association with indoor vs. outdoor work, hours worked per week, home type, or household size.

**Table 5:** Participant characteristics associated with *Ehrlichia* seropositivity among index workers.

Variable	Seropositive	Seronegative	P-value
N	25	87	
Sociodemographic characteristics			
Age (years), median (interquartile range)	47 (40-50)	40 (31-49)	0.17
Sex			0.59
Female	17 (68.0%)	54 (62.1%)	
Male	8 (32.0%)	33 (37.9%)	
Primary language			0.40
English	1 (4.0%)	8 (9.2%)	
Spanish	24 (96.0%)	79 (90.8%)	
Employment Industry			
Meat packing	10 (40.0%)	32 (36.8%)	0.77
Produce processing	2 (8.0%)	8 (9.2%)	0.85
Farming	13 (52.0%)	47 (54.0%)	0.86
Primarily indoor work	14 (56.0%)	56 (64.4%)	0.45
Average hours worked per week	41.7 ± 11.5	40.6 ± 9.9	0.65
Migrant farm worker	2 (8.0%)	7 (8.0%)	0.67
Home characteristics			
Home type			0.52
Single family	10 (40.0%)	21 (24.1%)	
Apartment	0 (0.0%)	1 (1.1%)	
Duplex/townhouse	0 (0.0%)	1 (1.1%)	
Trailer	14 (56.0%)	62 (71.3%)	
Dorm	1 (4.0%)	2 (2.3%)	
Household size	4.3 ± 1.7	4.3 ± 1.8	0.97

### Combined SFGR or *Ehrlichia* seroprevalence

Seropositivity for either SFGR or *Ehrlichia* was identified in 63 (31.3%) participants (S3 Table). Participants who were seropositive for either infection were older than participants who were seronegative (40.9 ± 18.2 vs. 29.2 ± 17.2, p < 0.001). Participants who spoke Spanish (OR 3.79, 95% CI 1.41-10.2, had diabetes mellitus or hypertension (OR 2.28, 95% CI: 1.12-4.61), or regularly drank alcohol (OR 5.86, 95% CI: 1.11-31.1) had higher odds of seropositivity for either infection; participants who attended school (OR 0.22, 95% CI: 0.09-0.51) had lower odds of seropositivity for either infection. These associations lost statistical significance when controlled for age.

Seven participants were seropositive for both SFGR and *Ehrlichia*. Four of these were farm workers.

### Household analysis

Out of 84 households, 26 (31.0%) had at least one member who was seropositive for SFGR, and 34 (40.5%) had at least one member who was seropositive for *Ehrlichia*. 50 households (59.5%) had at least one member who was seropositive for either infection. Household characteristics such as the presence of children (individuals less than 18 years of age), household size, type of home, and crowding (defined as the number of people in the house divided by the number of bedrooms) were not associated with having a seropositive member for either SFGR or *Ehrlichia* in the house. Among individual participants, having a household member who was seropositive for SFGR or *Ehrlichia* was associated with higher odds of seropositivity for the same infection, even after adjusting for age and accounting for household clustering of cases (adjusted OR [aOR] 8.26, 95% 3.27-20.90 for SFGR; aOR 11.24, 95% CI 4.24-29.80 for *Ehrlichia*) (Tables 2 and 4).

## Discussion

Among 201 food processing workers and their family members living in North Carolina during the fall of 2020, we identified SFGR and *Ehrlichia* IgG seropositivity in 14.9% and 19.9%, respectively. Seropositivity to at least one tickborne illness was identified in 31.3% of participants. Seroprevalence rates increased with age for both tickborne illnesses, although *Ehrlichia* was also common among children aged 10 years and younger, leading to a bimodal distribution for that pathogen. No other covariates in our analysis were significantly associated with SFGR or *Ehrlichia* seropositivity when adjusted for age. Having a seropositive household member was associated with higher risk of seropositivity for the same tickborne illness.

Limited data exist to compare our findings to prior seroprevalence studies in the North Carolina. A recent study of SFGR and *Ehrlichia* IgG antibodies among soldiers in North Carolina in 2019 showed seroprevalences of 12.0% and 3.8%, respectively [15]. Another cohort of 488 individuals in a population-based cohort from central North Carolina in 2017–2018 found SFGR and *Ehrlichia* seroprevalences of 17.1% and 8.6%, respectively [16]. In a 2016 study of 159 North Carolina outdoor workers, seroprevalence for *Rickettsia rickettsii* and *Ehrlichia chaffeensis* were 19% and 4%, respectively [8]. In our study, seroprevalence rates were similar to those from past studies for SFGR. However, the observed seroprevalence of *Ehrlichia* was markedly higher in our study at 19.9%. It is unclear whether this higher *Ehrlichia* seroprevalence is driven by geographic differences, occupational exposures, socioeconomic differences, or other factors. Some studies have reported rising *Ehrlichia* seroprevalence nationwide over the last few decades, thought to be in part due to expanding range of the lone star tick related to climate change [17,18], which could partially contribute to the higher seroprevalence observed in our study.

We found a marked association between older age and SFGR seropositivity, consistent with country-wide epidemiological data by the Centers for Disease Control and Prevention (CDC), which show a gradual increase in disease incidence by age group [19]. As tickborne antibodies can persist for years, this is an expected finding as older individuals have more time at risk. The CDC’s data show increasing *Ehrlichia* incidence with older age, although interestingly our study also found a high *Ehrlichia* seroprevalence in children under 10 years of age. Among the 25 children in our study age 10 years or younger, 6 (24.0%) were seropositive for *Ehrlichia*. High *Ehrlichia* seroprevalence in this population is concerning, as higher case fatality rate has been observed in children under 5 years of age [20]. If *Ehrlichia* incidence has indeed increased in the last decade, the bimodal distribution could represent a cohort effect [17,18]. As our study occurred during fall of 2020 when schools were closed due to the COVID-19 pandemic, it is possible that children were spending more time outside. This finding could also be explained by artifact in a small sample size.

We found that for both SFGR and *Ehrlichia*, individuals with a seropositive household member had a higher risk of seropositivity themselves for the same infection, even after accounting for household clustering of cases. Other studies have also shown clustering of symptomatic tickborne illnesses among family members and other close contacts, presumably due to shared environmental and behavioral exposures [21–24]. Workers may also carry ticks into the home on clothing. Thus, physicians should have a high degree of suspicion for tickborne disease among household contacts of recently diagnosed individuals who subsequently develop nonspecific symptoms.

Although the difference was not statistically significant, we find it notable that farm workers had higher SFGR seroprevalence (25.0%) than meat packing plant workers (16.7%) or produce processing workers (0.0%). Farm workers are highly exposed to ticks working outdoors, and inconsistent use of protective clothing and insect repellants as well as often substandard housing, particularly among migrant workers, may increase risk of tickborne illness in this group [2]. These individuals are also more vulnerable to severe disease from tickborne infections because of limited healthcare access, lack of insurance, language barriers, and poverty [4]. Although it is unclear why *Ehrlichia* seroprevalence did not vary by industry when SFGR seroprevalence did, differences in local tick population size and distribution or tick infection rates could contribute. Larger surveillance studies are needed to better assess tickborne infection risk among farm workers.

Our study did not demonstrate increased risk associated with outdoor work or specific occupation within food processing industries. However, our study does not exclude the possibility of occupational risk factors, primarily because, as it was conceived as a study of risk factors for SARS-COV-2 infection, it was not designed to address that question and does not include appropriate comparison groups such as workers in other industries to make such a conclusion. As we showed that having a seropositive household member increased an individual’s risk of seropositivity, household members of workers could have elevated risk compared to the general population. We also did not have data about whether family members had ever previously worked in the food processing industry themselves. In addition, our study is relatively small and underpowered to identify small differences in risk.

It is worth noting that our diagnostic testing did not distinguish between SFGR species. Although SFGR is typically associated with *Rickettsia rickettsii*, the causative agent of Rocky Mountain Spotted Fever, several other species have been found to cause clinical symptoms. In particular, *Rickettsia ambylommatis* is one of the most common species identified among lone star ticks in North Carolina, and some have theorized that it contributes to the high RMSF diagnosis rates in this region [25,26]. In addition, there is known cross-reactivity between *Ehrlichia* species (*E. chaffeensis, E. ewinigii, E. muris*).

Our study has several limitations. First, it was primarily designed to examine occupational risk factors for transmission of SARS-CoV-2 early in the COVID-19 pandemic, which may introduce bias. We did not collect data about many potential risk factors previously associated with tickborne illnesses such as pet ownership, self-reported tick exposures, or non-occupational outdoor activities [27–29]. As our study relies on self-report, we are likely missing data on medical comorbidities of our participants. Additionally, we cannot be certain that seropositive participants acquired either infection in North Carolina, although the vast majority of our participants had a fixed residence. Our study was relatively small and may be underpowered to detect small differences in risk between groups or result in overfitting of multivariate models. As our study focused on Latino communities in food processing industries in North Carolina, the external validity of our results to other populations may be limited. Finally, we do not have a comparison group or a robust existing body of literature to estimate tickborne disease seroprevalence in the general population in nearby geographic areas.

Overall, our study found high seroprevalence of SFGR and *Ehrlichia* antibodies among Latino food processing workers in North Carolina, raising concern for exposure to tickborne infections among food processing workers, particularly farmers, and the broader North Carolina population. Our study is one of the first to investigate seroprevalence of tickborne infections among Latino Americans, a population that remains vulnerable and often overlooked despite its growing size. We also identified a high *Ehrlichia* seroprevalence among young children, which could be a sentinel of a potentially increasing public health concern in North Carolina that warrants more surveillance. Given the nationwide rise of tickborne illness, larger epidemiological and entomological surveillance studies are needed to better quantify geographic trends in tickborne illness and identify occupational and socioeconomic risk factors.

## Supporting information

S1 Table Sensitivity analysis of participant characteristics associated with SFGR seropositivity among index workers and household members. (DOCX)

S2 Table Sensitivity analysis of participant characteristics associated with SFGR seropositivity among index workers and household members. (DOCX)

S3 Table Participant characteristics associated with either SFGR or *Ehrlichia* seropositivity among index workers and household members. (DOCX)

S1 Checklist Inclusivity in global research. (DOCX)
