# Supplementary material for: Seroprevalence of spotted fever rickettsiosis and ehrlichiosis among food processing workers and their families in Latino communities in North Carolina
Source: PLOS Glob Public Health. 2025 Sep 2;5(9):e0005116. doi: 10.1371/journal.pgph.0005116 (PMC12404551; doi:10.1371/journal.pgph.0005116)
Supplement: S2 Table — (DOCX) [file pgph.0005116.s002.docx]

**S1 Table. Sensitivity analysis of participant characteristics associated with SFGR seropositivity among index workers and household members.** Equivocal SFGR seropositivity results (n=6) were considered positive. SFGR: Spotted Fever Group Rickettsiosis

| **Variable** | **Seropositive** | **Seronegative** | **P-value** | **OR** | **aOR^a^** |
| --- | --- | --- | --- | --- | --- |
| N | 36 | 159 |  |  |  |
|  |  |  |  |  |  |
| Sociodemographic characteristics |  |  |  |  |  |
| Age (years), median (interquartile range) | 45 (36-56.5) | 31 (13-44) | <0.001 | **1.06 (1.03-1.09)** |  |
| Sex |  |  | 0.51 | 0.78 (0.37-1.64) |  |
| Male | 22 (61.1%) | 91 (57.2%) |  |  |  |
| Female | 14 (38.9%) | 74 (46.5%) |  |  |  |
| Primary language |  |  | 0.06 | 3.07 (0.89-10.6) |  |
| English | 3 (8.3%) | 36 (22.6%) |  |  |  |
| Spanish | 33 (91.7%) | 129 (81.1%) |  |  |  |
|  |  |  |  |  |  |
| Employment status |  |  | 0.64 | 1.09 (0.70-1.67) |  |
| Currently employed full-time | 22 (61.1%) | 74 (46.5%) |  |  |  |
| Currently employed part-time | 2 (5.6%) | 13 (8.2%) |  |  |  |
| Unemployed | 10 (27.8%) | 25 (15.7%) |  |  |  |
| Other | 0 (0.0%) | 1 (0.6%) |  |  |  |
| Medical comorbidities |  |  |  |  |  |
| Diabetes mellitus or hypertension | 13 (36.1%) | 28 (17.6%) | 0.007 | **2.89 (1.30-6.42)** | 0.74 (0.26-2.10) |
| Ever smoker | 7 (19.4%) | 11 (6.9%) | 0.02 | **3.25 (1.16-9.08)** | 2.05 (0.68-6.18) |
| Regular alcohol use^b^ | 3 (8.3%) | 4 (2.5%) | 0.08 | 3.66 (0.78-17.1) |  |
| Social characteristics |  |  |  |  |  |
| School attendance | 2 (5.6%) | 56 (35.2%) | 0.001 | **0.12 (0.03-0.51)** | 0.29 (0.04-2.00) |
| Recent travel^c^ | 1 (2.8%) | 13 (8.2%) | 0.29 | 0.34 (0.04-2.70) |  |

^a^Adjusted for age

^b^Defined as routinely drinking 7 or more drinks per week

^c^Defined as travel within the last 3 weeks in which at least one night was spent away from home

**S2 Table. Sensitivity analysis of participant characteristics associated with SFGR seropositivity among index workers and household members.** Equivocal SFGR seropositivity results (n=6) were excluded. SFGR: Spotted Fever Group Rickettsiosis

| **Variable** | **Seropositive** | **Seronegative** | **P-value** | **OR** | **aOR^a^** |
| --- | --- | --- | --- | --- | --- |
| N | 30 | 159 |  |  |  |
|  |  |  |  |  |  |
| Sociodemographic characteristics |  |  |  |  |  |
| Age (years), median (interquartile range) | 45 (35-55) | 31 (13-44) | <0.001 | **1.05 (1.03-1.08)** |  |
| Sex |  |  | 0.41 | 0.71 (0.32-1.59) |  |
| Male | 19 (63.3%) | 91 (57.2%) |  |  |  |
| Female | 11 (36.7%) | 74 (46.5%) |  |  |  |
| Primary language |  |  | 0.054 | 3.91 (0.89-17.2) |  |
| English | 2 (6.7%) | 36 (22.6%) |  |  |  |
| Spanish | 28 (93.3%) | 129 (81.1%) |  |  |  |
|  |  |  |  |  |  |
| Employment status |  |  | 0.55 | 1.03 (0.64-1.65) |  |
| Currently employed full-time | 19 (63.3%) | 74 (46.5%) |  |  |  |
| Currently employed part-time | 1 (3.3%) | 13 (8.2%) |  |  |  |
| Unemployed | 8 (26.7%) | 25 (15.7%) |  |  |  |
| Other | 0 (0.0%) | 1 (0.6%) |  |  |  |
| Medical comorbidities |  |  |  |  |  |
| Diabetes mellitus or hypertension | 8 (26.7%) | 28 (17.6%) | 0.18 | 1.86 (0.75-4.63) |  |
| Ever smoker | 6 (20.0%) | 11 (6.9%) | 0.02 | **3.36 (1.14-9.95)** | 2.24 (0.71-7.07) |
| Regular alcohol use^b^ | 3 (10.0%) | 4 (2.5%) | 0.04 | 4.47 (0.95-21.1) |  |
| Social characteristics |  |  |  |  |  |
| School attendance | 2 (6.7%) | 56 (35.2%) | 0.003 | **0.14 (0.03-0.63)** | 0.44 (0.06-3.23) |
| Recent travel^c^ | 0 (0.0%) | 13 (8.2%) | 0.12 | - |  |

^a^Adjusted for age

^b^Defined as routinely drinking 7 or more drinks per week

^c^Defined as travel within the last 3 weeks in which at least one night was spent away from home

**S3 Table. Participant characteristics associated with either SFGR or *Ehrlichia* seropositivity among index workers and household members.** SFGR: Spotted Fever Group Rickettsiosis

| **Variable** | **Seropositive** | **Seronegative** | **P-value** | **OR** | **aOR^a^** |
| --- | --- | --- | --- | --- | --- |
| N | 63 | 138 |  |  |  |
|  |  |  |  |  |  |
| Sociodemographic characteristics |  |  |  |  |  |
| Age (years), median (interquartile range) | 44 (31-52) | 30 (13-43) | <0.001 | **1.04 (1.02-1.06)** |  |
| Sex |  |  | 0.89 | 1.04 (0.57-1.90) |  |
| Male | 35 (55.6%) | 78 (56.5%) |  |  |  |
| Female | 28 (44.4%) | 60 (43.5%) |  |  |  |
| Primary language |  |  | 0.005 | **3.79 (1.41-10.2)** | 1.80 (0.59-5.46) |
| English | 5 (7.9%) | 34 (24.6%) |  |  |  |
| Spanish | 58 (92.1%) | 104 (75.4%) |  |  |  |
|  |  |  |  |  |  |
| Employment status |  |  | 0.47 | 1.15 (0.79-1.68) |  |
| Currently employed full-time | 34 (54.0%) | 62 (44.9%) |  |  |  |
| Currently employed part-time | 4 (6.3%) | 11 (8.0%) |  |  |  |
| Unemployed | 16 (25.4%) | 19 (13.8%) |  |  |  |
| Other | 0 (0.0%) | 1 (0.7%) |  |  |  |
| Medical comorbidities |  |  |  |  |  |
| Diabetes mellitus or hypertension | 19 (30.2%) | 22 (15.9%) | 0.02 | **2.28 (1.12-4.61)** | 0.83 (0.34-2.04) |
| Ever smoker | 9 (14.3%) | 9 (6.5%) | 0.09 | 2.28 (0.86-6.06) | 1.53 (0.55-4.26) |
| Regular alcohol use^b^ | 5 (7.9%) | 2 (1.4%) | 0.02 | **5.86 (1.11-31.1)** | 4.27 (0.79-23.0) |
| Social characteristics |  |  |  |  |  |
| School attendance | 7 (11.1%) | 51 (37.0%) | <0.001 | **0.22 (0.09-0.51)** | 0.43 (0.14-1.30) |
| Recent travel^c^ | 0 (0.0%) | 14 (10.1%) | 0.01 | - |  |

^a^Adjusted for age

^b^Defined as routinely drinking 7 or more drinks per week

^c^Defined as travel within the last 3 weeks in which at least one night was spent away from home
